# PI by NMR: Probing CH–π Interactions in Protein–Ligand Complexes by NMR Spectroscopy

**DOI:** 10.1002/anie.202003732

**Published:** 2020-07-15

**Authors:** Gerald Platzer, Moriz Mayer, Andreas Beier, Sven Brüschweiler, Julian E. Fuchs, Harald Engelhardt, Leonhard Geist, Gerd Bader, Julia Schörghuber, Roman Lichtenecker, Bernhard Wolkerstorfer, Dirk Kessler, Darryl B. McConnell, Robert Konrat

**Affiliations:** ^1^ Christian Doppler Laboratory for High-Content Structural Biology and Biotechnology Department of Structural and Computational Biology Max Perutz Labs University of Vienna Campus Vienna Biocenter 5 1030 Vienna Austria; ^2^ Boehringer Ingelheim RCV GmbH & Co. KG Dr. Boehringer Gasse 5–11 1121 Vienna Austria; ^3^ Institute of Organic Chemistry University of Vienna Währingerstraße 38 1090 Vienna Austria

**Keywords:** CH–π interactions, medicinal chemistry, NMR spectroscopy, protein–ligand interactions, structure-based drug design

## Abstract

While CH–π interactions with target proteins are crucial determinants for the affinity of arguably every drug molecule, no method exists to directly measure the strength of individual CH–π interactions in drug–protein complexes. Herein, we present a fast and reliable methodology called PI (π interactions) by NMR, which can differentiate the strength of protein–ligand CH–π interactions in solution. By combining selective amino‐acid side‐chain labeling with ^1^H‐^13^C NMR, we are able to identify specific protein protons of side‐chains engaged in CH–π interactions with aromatic ring systems of a ligand, based solely on ^1^H chemical‐shift values of the interacting protein aromatic ring protons. The information encoded in the chemical shifts induced by such interactions serves as a proxy for the strength of each individual CH–π interaction. PI by NMR changes the paradigm by which chemists can optimize the potency of drug candidates: direct determination of individual π interactions rather than averaged measures of all interactions.

## Graphical abstract

**CH–π interactions** are crucial determinants for the affinity of arguably every drug molecule. PI by NMR can differentiate the strength of protein–ligand CH–π interactions in solution. By combining selective amino‐acid side‐chain labeling with ^1^H‐^13^C NMR, CH–π interactions can be identified based solely on ^1^H chemical shift values. This information serves as a proxy for the strength of each individual CH–π interaction.

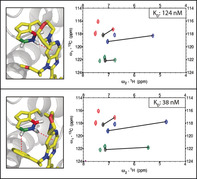

## Introduction

Fragment‐based drug discovery (FBDD)^1^ is based on the continuous optimization of weakly binding hits derived from initial screening methods towards highly selective and potent compounds that target proteins implicated in various types of disease. This process relies on the tuning of weak reversible interactions, the most important of them being van der Waals, electrostatic, and hydrogen bonding interactions.^2^ A special case of weak molecular forces that fine‐tune molecular recognition events involves the interaction of the π‐electron cloud of aromatic ring systems with aromatic or aliphatic hydrocarbons. In the literature, this interaction is often termed CH–π interaction or CH–π hydrogen bond,^3^ or simply classified as a type of hydrophobic interaction.^4^ Here the aliphatic or aromatic CH group acts as a hydrogen donor (a soft acid) and the π system as the hydrogen acceptor (a weak base). According to theoretical studies in the gas phase, this interaction is weak and mainly dominated by dispersive forces with a small electrostatic component.^5^ For non‐activated CH groups, the interaction energy is about −1.5 kcal mol^−1^ and for activated, more acidic CH‐groups, like acetylene or chloroform, the interaction becomes more favorable, owing to an increase in electrostatic character.^5^ Reviews on interactions involving aromatic π systems in general3a, 3b, 6 and CH–π interactions in particular3c have been published. However, a clear definition of interactions involving π‐systems remains a subject of debate.^6^, ^7^ CH–π interactions are major modulators of affinity and selectivity in protein–ligand complexes.^4^, ^8^ This is evidenced by the high relative occurrence of aromatic amino acids in drug pockets^9^ and the large number of aliphatic donor groups implicated in protein–ligand aromatic interactions.^4^ Most drugs contain one or more aromatic ring system with an average of 1.8 in recently marketed drugs.^10^

Present‐day drug‐discovery programs rely on the modification of weak initial binders by optimizing van der Waals interactions by shape complementarity, electrostatic interactions by charge matching, and inferring H‐bond donor or acceptor groups from structural information. On the contrary, no method currently exists to assess the beneficial or detrimental effects of CH–π interactions to the overall affinity of a protein–ligand complex. Their impact can only be inferred indirectly through global measures of affinity (*K*_D_) or derived from geometrical observations versus historical statistical distributions (for example, X‐ray crystallography). Having a method to directly gauge the strength of individual CH–π interactions rather than averaged measures of all interactions would greatly benefit drug‐potency optimization.

NMR spectroscopy is uniquely suited to extract site‐specific information about molecular interactions at atomic resolution and is routinely used to guide drug‐development processes.^11^ Although a first example has been given with the detection of weak J‐couplings between nuclei involved in methyl–π interactions within proteins,^12^ a general detection strategy for CH–π interactions in protein–ligand complexes is still missing. It is important to note that suitably resolved NMR spectra can only be obtained using selective labeling, otherwise ^1^ *J*_CC_ (strong) scalar coupling effects lead to substantially lower signal‐to‐noise. The precise and accurate measurement of chemical‐shift information is considerably facilitated by the availability of suitably labeled aromatic amino‐acid side‐chains for NMR detection.^13^ Specific tryptophan labeling can be achieved by providing either the α‐ketoacid derivative of tryptophan, namely indolepyruvate, or simply anthranilic acid.^14^ Different isotopologues of these compounds can be prepared by efficient multistep organic synthesis allowing for unique isotope‐labeling patterns in aromatic amino acids and very sensitive NMR detection schemes.^14^

Herein, we show that the combination of amino‐acid‐labeling strategies and sensitive ^1^H‐^13^C protein NMR spectroscopy provides unprecedented insight into the details of CH–π interactions and their relevance for protein–ligand complexes. Monitoring the induced change in chemical shifts of protein side‐chain protons engaged in a CH–π interaction with aromatic ligand moieties allows the identification of favorable CH–π interactions relevant for binding. The relationship between the observed chemical‐shift change and beneficial stacking interactions can be used as a direct read‐out for assessing the quality of individual, stabilizing CH–π interactions, thereby guiding further lead optimization.

## Results

### Direct Detection of CH–π Interactions by NMR Spectroscopy

CH–π interactions are crucial determinants in protein–ligand interactions. They comprise aliphatic sp^3^‐hybridized groups, most prominently leucine, valine, isoleucine, and alanine CH_3_ groups, and sp^2^‐hybridized CH groups of aromatic amino acids,^4^ among which tryptophan has the highest preference factor, followed by histidine and phenylalanine.^9^ While the general applicability of this method applies to all amino acids able to act as a hydrogen donor (aromatic and aliphatic), this work will focus on the example of tryptophan.

**Figure 1:**
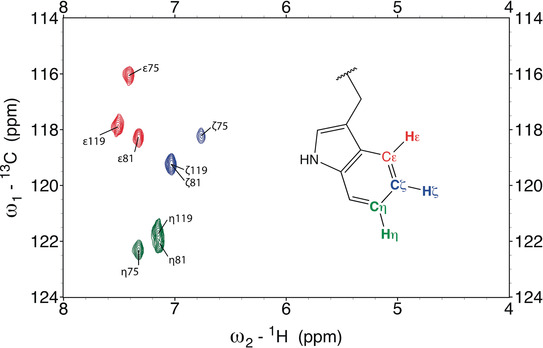
Overlay of two ^1^H‐^13^C HSQC spectra of Brd4‐BD1 selectively labeled either at positions *η* (green) and *ϵ* (red) or at position *ζ* (blue). For each set of spin systems, three signals are observed, which correspond to the three tryptophan residues (Trp75, Trp81 and Trp119) present in Brd4‐BD1. Note that signals for Trp81‐*ζ* and Trp119‐*ζ* (blue cross peaks) at position ^1^H: 7.0 ppm/ ^13^C: 119.2 ppm, as well as Trp81‐*η* and Trp119‐*η* (green cross peaks) at position ^1^H: 7.1 ppm/ ^13^C: 122.0 ppm, overlap.

Here we employed precursor‐assisted labeling of tryptophan *η* (eta), *ϵ* (epsilon), and *ζ* (zeta) carbons by supplementing standard minimal media with selectively labeled anthranilic acid, a metabolic precursor of tryptophan.^14^ By combining two precursors, one labeled at positions *η* and *ϵ*, the other at position *ζ*, we reduce the number of NMR observables to a total of three signals per tryptophan. The binding domain 1 of the bromodomain‐containing protein 4 (Brd4‐BD1) that recognizes acetylated lysine residues,^25^ was chosen as model system, due to the availability of a large set of in‐house protein–ligand co‐crystal structures. Brd4‐BD1 contains three tryptophans, yielding a total of nine signals (Figure 1).

In the approach presented here the experimental “read out” to probe CH–π interactions is the proton chemical‐shift perturbation (CSP) and line broadening of the isolated ^1^H‐^13^C spin‐pair resonances induced by ligand binding. Significant ^1^H chemical‐shift changes are induced by the bound ligand, provided that a CH–π interaction exists. Line broadening can occur due to reversible ligand binding (in case of μm *K*_D_s) and in cases of multiple binding modes. Contributions from reversible binding can be excluded for a fully ligand‐saturated protein.^15^ In the following, we show that, in the case of Brd4‐BD1 ligand complexes, the CH‐groups of Trp81 are sensitive reporters for favorable CH–π contacts and can be easily monitored with 2D ^1^H‐^13^C HSQC NMR experiments. In order to provide a comprehensive assessment, we selected a set of 13 ligands (Supporting Information, Table S1) for which both crystal structures and affinity data were available. The selection of ligands covers representative binding modes observed in typical drug‐development programs.

**Figure 2:**
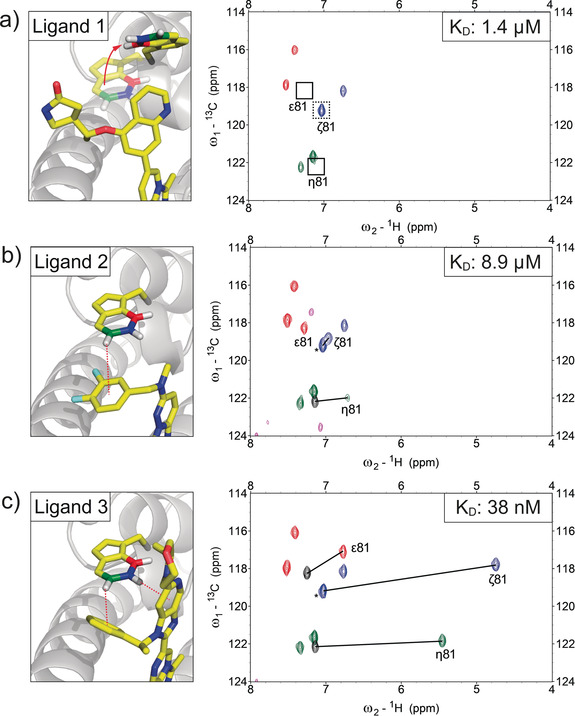
NMR probing of protein–ligand CH–π interactions. X‐ray crystal structures of ligands 1 to 3 bound to Brd4‐BD1 and ^1^H‐^13^C HSQC overlays of protein selectively labeled at tryptophan bound to the respective ligands. Missing signals are indicated with black boxes. Black lines indicate the CSP induced by the ligand. Ligand signals are shown in magenta. Black peaks correspond to the apo protein signals of the shifting resonances. In the case of Trp81‐*ζ*, the apo‐signal is overlapped by Trp119‐*ζ* (denoted by *). a) Complete loss of all Trp81 signals due to an extensive global conformational rearrangement induced upon ligand binding. The loss of signal for Trp81‐*ζ* is evidenced by the signal reduction of the peak highlighted with a dashed box. b) Ligand 2 shows site‐selective line‐broadening of the interacting *η*‐CH pair with Δ*ω*H−*η*=0.43 ppm. c) Favorable stacking interactions of ligand 3 lead to extensive CSPs (Δ*ω*H−*ζ*=2.3 ppm & Δ*ω*H−*η*=1.69 ppm).

Figure 2 shows the overlay of the ^1^H‐^13^C HSQC spectra of selectively *ζ* (blue) and *η*/*ϵ* (red/green) labeled Brd4‐BD1 bound to various ligands representing different binding modes. Three cases can be discriminated: i) the complete loss of signals due to a global conformational rearrangement of the interacting tryptophan (Figure 2 a), ii) line broadening due to intermediate exchange effects (Figure 2 b), and iii) substantial CSPs due to well‐defined CH–π interactions (Figure 2 c).

Reversible binding of ligand 1 to Brd4‐BD1 (Figure 2 a) leads to the loss of all three Trp81 ^1^H‐^13^C signals, a pronounced case of conformational‐exchange‐induced line broadening^16^ due to a substantial structural rearrangement of the tryptophan side‐chain. Although seemingly disappointing, this observation might help with clustering ligands according to their mode of binding.

Figure 2 b shows exemplary data for ligands with moderate binding affinities and site‐selective line‐broadening effects (only the *η* ^1^H‐^13^C signal is affected). Ligand 2 displays a significantly broadened NMR signal with a moderate upfield shift of the interacting *η* proton Δ*ω*H−*η*=0.43 ppm (Figure 2 b, right). The observed line broadening is partly due to its low binding affinity (complete ligand saturation cannot be achieved), but also other effects might contribute (for example, residual mobility in the bound state and alternative binding modes).^15^ It is important to note that despite the lower binding affinity, the observed CSPs can be quantitatively related to binding geometry.

Ligand 3 (Figure 2 c) is representative of ligands with optimal stacking geometries that lead to substantial upfield shifts of the involved tryptophan CH protons upon ligand binding. The presence of aromatic ring systems above the *ζ*‐ and the *η*‐CH groups of Trp81 leads to substantial upfield shifts of Δ*ω*H−*η*=1.69 ppm and Δ*ω*H−*ζ*=2.3 ppm, respectively.

### Geometric Parameters of the CH–π Interface

Encouraged by the large CSPs for protein CH groups in contact with aromatic π systems, we set out to analyze the underlying geometric dependencies. Protein chemical shifts have already been successfully used in some instances to orient ligands in the binding pocket.^17^ The observed chemical‐shift changes are due to ring current effects of ligand aromatic rings and electric‐field effects caused by charged groups.^18^ Protons in spatial proximity to an aromatic ring system experience differential effects from strong shielding to weak deshielding, depending on the orientation of the aromatic ring relative to the protons being affected.^19^

**Figure 3:**
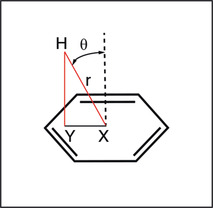
Geometric parameters extracted from X‐ray crystal structures are the proton‐to‐ring‐center distance (*r*, H–X), the proton‐to‐plane distance (H–Y), the angle (*θ*) between the ring normal through the aromatic center (X) and the proton‐to‐ring‐center vector.

In order to correlate this orientation dependence with proton CSP values of the interaction, we analyzed our set of 13 ligands. For all ligands X‐ray, as well as NMR data was available. We apply a model first introduced by Pople^20^ where the center of the aromatic ring is treated as a point dipole inducing a magnetic field given by the standard dipole equation:
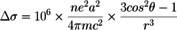



where Δ*σ* is the change in the isotropic nuclear shielding constant in ppm, *n* is the number of circulating electrons (*n*=6 for all ligands discussed in this work), *e* is the elementary charge in Franklin, *a* is the radius of the aromatic ring (1.39×10^−08^ cm), *m* is the electron mass in gram, *c* is the speed of light in cm s^−1^, *θ* is the angle between the ring normal through the aromatic center (X) and the proton to ring center vector in rad, and *r* is the distance from the proton to the ring center (H–X) in cm (Figure 3).15c

**Figure 4:**
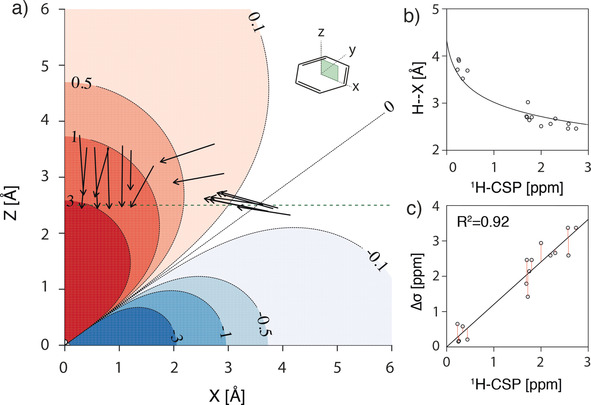
a) Black arrows represent the projections of the CH bond vectors for tryptophan CH groups interacting with ligand aromatic ring systems of ligands 2 to 13 onto the *x*−*z*=*y*−*z* plane of the calculated isotropic shielding surface Δ*σ*(*r*,*θ*) in ppm. Individual conformational parameters are shown in Table S1 in the Supporting Information. The green dashed line corresponds to the most frequently found H–Y distance of 2.5 Å. b) Comparison between calculated chemical shifts and their dependence on the proton‐to‐ring‐center distance (H–X). In the calculation (solid line) the H–Y distance was set to 2.5 Å. c) Correlation of CSP with the calculated nuclear shielding constant Δ*σ*. Calculated values for Δ*σ* from X‐ray crystal structures are well reproduced by experimental data (R^2^=0.92).

The geometric parameters *r* and *θ* were extracted from X‐ray crystal structures based on the model depicted in Figure 3 (see also Table S1 in the Supporting Information). Interestingly, the proton‐to‐plane distance (H–Y) showed only small variations between 2.4 to 2.9 Å, except for ligand 12 with a H–Y distance of 3.27 Å, with a CSP value of 0.23 ppm, which is the lowest among all ligands studied (Figure 4 a). More variability was found for the proton‐to‐ring‐center distances (H–X), which were between 2.5 and 4.0 Å (Figure 4 b). The theoretical CSP values derived from Equation 1 are in very good agreement with experimental CSP values (ranging from 0.23 ppm to 2.74 ppm, see Table S1 in the Supporting Information; Figure 4 c). The slight deviation from the theoretically expected slope of 1 is due to limitations of the Pople model. However, if properly parameterized, the precision of the point‐dipole model is comparable to more sophisticated models as has been reported before.15c, 21 We also want to emphasize that, although the experimental data set also contains ligands with moderate binding affinities, the correlation to predicted X‐ray‐crystal‐structure‐derived CSP values is equally good. Thus, even in cases where the binding affinity is only moderate and far from optimal, relevant structural information about the ligand binding mode can be extracted. The largest CSP values are obtained when the H–X distance is minimal, equivalent with stacking of the CH‐donor directly above the ring center. Thus, CSP values are very efficient sensors to probe proton‐to‐ring‐center orientations (Figure 3). It is very instructive to compare the relative Trp81 orientations in the different protein–ligand complexes and their relationship to proton‐to‐ring‐center distances (Figure 4 a). Small H–X distances are found for T‐stacked binding modes. For larger H–X distances, the tryptophan is shifted laterally and tilted relative to the aromatic ring system, resembling a parallel displaced stacking mode.

### Do Large Upfield CSPs Correlate with Favourable CH–π Interactions?

Given the good agreement between favorable stacking geometries and the observed change in the proton chemical shift of the interacting protein CH group, we took a closer look at the energetic details of the interaction. Interactions between an aromatic acceptor group with aromatic sp^2^ (benzene–benzene), as well as aliphatic sp^3^ donor groups (for example, benzene–methane) have been analyzed with both theoretical^5^, ^22^ and experimental methods.^23^ According to calculations for benzene–benzene^22^ and benzene–methane interactions,^5^ the most favourable configuration would put the donor proton directly on top of the aromatic ring center at a distance of approximately 2.5 Å, consistent with our experimental data for donor protons stacking on top of ligand ring systems.

**Figure 5:**
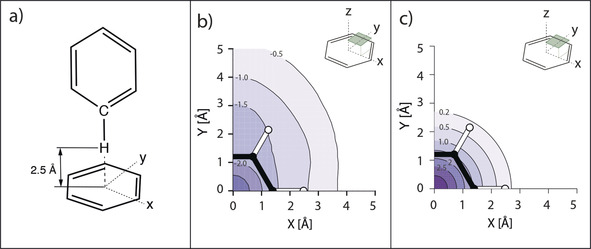
a) Configuration for the calculation of Δ*H* values. The CH‐donor group of one benzene molecule was placed perpendicularly at a H–Y distance of 2.5 Å with respect to the interacting aromatic acceptor group of the second benzene ring. b) Energy surface for an orthogonal scan of the benzene–benzene interaction at a distance of 2.5 Å in units of kcal mol^−1^. Asymmetry in calculated energies for *x*‐/*y*‐axes is mainly caused by the constant orientation of the CH‐donating benzene ring along the *y*‐axis along with the contribution of *ortho* hydrogens to the interaction. c) Calculated isotropic shielding values (Δ*σ*) for the same benzene–benzene interaction at a distance of 2.5 Å in units of ppm.

We therefore employed DFT calculations for the interaction energies on the T‐shaped stacking conformation by scanning the benzene surface in an orthogonal fashion at the optimal proton‐to‐ring‐plane distance (H–Y) of 2.5 Å (Figure 5 a). Figure 5 b shows the resulting energy heatmap for the interaction of the CH‐donor group of one benzene group with the π system of the second benzene group as acceptor system. The DFT calculations revealed an interaction energy surface minimum (−2.86 kcal mol^−1^) when the donor group is stacking directly on top of the ring center (H–X=H–Y=2.5 Å; *θ*=0°). Comparing the interaction energy surface (Figure 5 b) with the calculated isotropic shielding constants, Δ*σ* (Figure 5 c) shows that pronounced shielding (greater than 0.2 ppm) of a protein donor proton is indicative of a favorable interaction energy contribution (approximately 1.0 kcal mol^−1^). We thus conclude that the observed correlation between CSPs and interaction energies can serve as a proxy for beneficial CH–π interactions stabilizing protein–ligand complexes.

**Figure 6:**
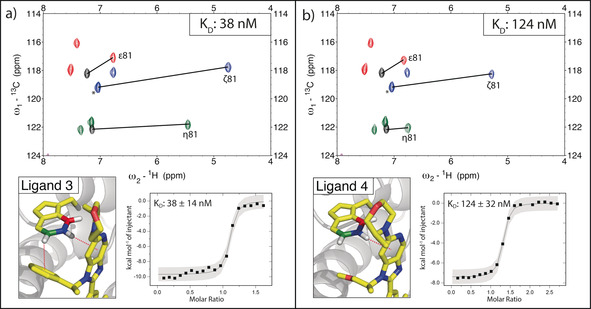
X‐ray crystal structure of the matched pair ligands 3 and 4 bound to Brd4‐BD1 with ITC data and ^1^H‐^13^C HSQC overlays of protein selectively labeled at tryptophan bound to the respective ligands. Black lines indicate the experienced CSP upon ligand addition. Black peaks correspond to the apo‐protein signal. In the case of Trp81‐*ζ* the apo‐signal is overlapped by Trp119‐*ζ* (denoted by *) a) Favorable stacking interactions of ligand 3 lead to extensive CSPs. b) Ligand 4 is structurally identical to ligand 3 except for the interaction interface with Trp81‐*η* (phenyl vs. methoxy propyl). The favorable stacking interaction of ligand 4 leads to a *ζ*‐proton shift of 1.75 ppm. The presence of an additional CH‐π interaction for ligand 3 over ligand 4 is partly reflected in the difference in binding enthalpy and affinity between the two ligands.

To demonstrate the relevance of CSPs for the identification of favourable CH–π interactions and their contribution to binding affinity, we analyzed a matched ligand pair (ligands 3 and 4), which are structurally identical except for the interaction interface with Trp81‐*η* (methoxy propyl vs. phenyl). ITC measurements were carried out to extract binding affinities and thermodynamic parameters (Figure 6).

The affinity of ligand 3 is significantly higher (*K*_D_: 38 nm; Δ*H*: −9.2 kcal mol^−1^) due to an additional CH–π interaction present compared to ligand 4 (*K*_D_: 124 nm; Δ*H*: −7.5 kcal mol^−1^). The experimental difference in binding enthalpy amounts to −1.7 kcal mol^−1^, which compares well with theoretical calculations for a perfect benzene–benzene T‐shaped orientation (−2.45 kcal mol^−1^).^22^ Interestingly, chemical‐shift changes observed for all (*ζ*, *η*, and *ϵ*) ^1^H‐^13^C Trp signals are consistently higher in ligand 3, thus providing independent evidence for an improved binding interaction. Although other contributions, for example, differential solvent effects,^24^ might play a role, our measurements highlight that the robust improvement in overall binding affinity for ligand 3 can be readily observed and (at least in part) attributed to improved CH–π interactions.

## Discussion

Efforts to exploit non‐covalent interactions in protein–ligand complexes to improve potency and selectivity of drugs would greatly benefit from the availability of information‐rich experimental techniques to probe and quantify these interactions, ideally with atomic resolution. A prominent case of non‐covalent forces that fine‐tune molecular recognition events involves the interaction between aromatic ring systems and aromatic or aliphatic hydrocarbons, often termed CH–π interaction or CH–π hydrogen bond. Despite the undisputed relevance of this interaction, experimental demonstrations have been scarce due to technical limitations. Although X‐ray crystallography can provide detailed structural information, an unambiguous demonstration of the existence of stabilizing CH–π interactions in solution is not straightforward. Here we show that our approach, PI by NMR, probes individual CH–π interactions and enables medicinal chemists to move away from coarse‐grained methods quantifying global measures of affinity to a site‐specific interaction‐based design strategy.

The exquisite sensitivity of the chemical shift to subtle changes of the chemical environment makes NMR the method of choice to probe small variations of chemical environments upon, for example, ligand binding to a protein target. Although this feature is widely known and generally accepted, applications to macromolecular systems typically found in drug‐discovery programs were limited due to experimental limitations. Among other reasons, the overwhelming NMR spectral complexity of proteins together with limited sensitivity and unwanted scalar coupling effects were hampering applications. We previously described that appropriate selective precursors for aromatic residue isotope labeling can be synthesized and used in bacterial cell cultures to selectively label defined positions in the aromatic amino‐acids phenylalanine, tyrosine, tryptophan, and histidine.^26^

The application of these labeling techniques to study CH–π interactions in protein–ligand complexes offers exciting possibilities in drug‐design programs. Importantly, the technique is applicable to protein–ligand systems covering a wide range of binding affinities from mm to nm, indicating the potential for structure‐based drug‐design programs. We anticipate that the broader implementation of our approach will impact future drug‐development programs in several ways. First, simply tracking the extent of chemical shifts induced by ligand binding allows the identification of favorable CH–π interactions relevant for binding, thereby guiding further lead optimization. Monitoring whether the optimal binding modes are retained during compound optimization is consequently straightforward and only requires comparison of the ligand‐induced CSPs. Second, numerous chemical scaffolds are found in early stages of FBDD programs. Selection of the most promising fragments suitable for subsequent lead optimization is still a daunting task. Observation of the chemical‐shift changes induced by aromatic ligand moieties is indicative of favorable CH–π interactions and may constitute an important fragment selection criterion. Finally, the growing arsenal of selectively labeled precursors covering all relevant protein CH‐donor groups will allow unique pharmacophore features to be addressed in hitherto unexplored protein binding pockets, even in the absence of X‐ray crystallographic data. We are currently building up on the premise of using fragment‐induced CSP information from side‐chain‐labeled protein to guide a chemical design strategy in order to optimally position ligand aromatic rings within the binding site of the protein target of interest.

We anticipate that the ease of implementation and high spatial resolution of PI by NMR will change the paradigm by which chemists optimize drug potency. The possibility to specifically optimize CH–π interactions in protein–ligand complexes clearly has the potential to transform how we design molecular therapeutics.

## Conflict of interest

The authors declare no conflict of interest.

## Supporting information

As a service to our authors and readers, this journal provides supporting information supplied by the authors. Such materials are peer reviewed and may be re‐organized for online delivery, but are not copy‐edited or typeset. Technical support issues arising from supporting information (other than missing files) should be addressed to the authors.

Supplementary
