# Supplementary material for: PI by NMR: Probing CH–π Interactions in Protein–Ligand Complexes by NMR Spectroscopy
Source: Angew Chem Int Ed Engl. 2020 Jul 15;59(35):14861–8. doi: 10.1002/anie.202003732 (PMC7496880; doi:10.1002/anie.202003732)
Supplement: Supplementary file 1 — Supplementary [file ANIE-59-14861-s001.pdf]

Angewandte Chemie
Eine Zeitschrift der Gesellschaft Deutscher Chemiker

Supporting Information

PI by NMR: Probing CH–π Interactions in Protein–Ligand Complexes by NMR Spectroscopy

Gerald Platzer, Moriz Mayer,* Andreas Beier, Sven Brüschweiler, Julian E. Fuchs, Harald Engelhardt, Leonhard Geist, Gerd Bader, Julia Schörghuber, Roman Lichtenecker, Bernhard Wolkerstorfer, Dirk Kessler, Darryl B. McConnell, and Robert Konrat*

anie_202003732_sm_miscellaneous_information.pdf

Table 1: Structures, chemical shifts and geometric parameters

Ligand	Structure	Trp81 CSP H(ε) [ppm]	Trp81 CSP H(ζ) [ppm]	Trp81 CSP H(η) [ppm]	CH donor	H--X [Å]	H--Y [Å]	θ [°]	Δσ [ppm]
**1**	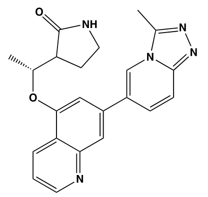	global conformational exchange							
**2**	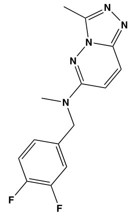	0.04	0.10	0.43 (broad)	Trp81-η	3.72	2.60	45.7	0.23
**3**	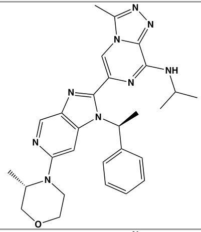	0.56	2.30	1.69	Trp81- ζ	2.70	2.68	7.0	2.58
					Trp81-η	2.75	2.47	26.1	1.77
**4**	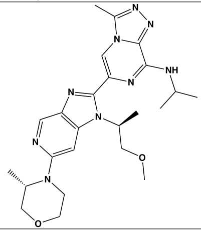	0.50	1.75	0.39	Trp81- ζ	2.68	2.47	22.8	2.09
**5**	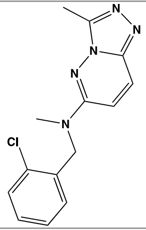	0.10	0.23	exch. br.	Trp81-η	3.76<br>4.02	2.64<br>2.47	45.4<br>52.1	0.23<br>0.05
**6**	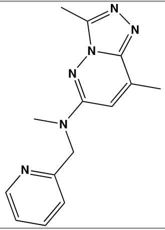	0.03	0.08	exch. br.	Trp81-η	3.66	2.62	44.3	0.28
**7**	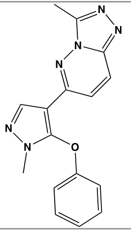	0.03	0.0	0.25 (broad)	Trp81-η	3.96	2.73	46,4	0.18

Ligand	Structure	Trp81 CSP H(ε) [ppm]	Trp81 CSP H(ζ) [ppm]	Trp81 CSP H(η) [ppm]	CH donor	H--X [Å]	H--Y [Å]	θ [°]	Δσ [ppm]
**8**	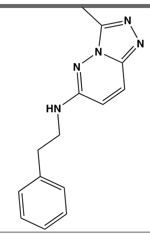	0.00	0.04	0.44 (broad)	Trp81-η	3.52	2.90	34.5	0.62
**9**	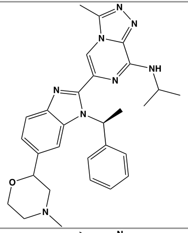	0.60<br>0.65	2.57<br>2.74	1.73<br>1.82	Trp81- ζ	2.46	2.44	7.3	3.40
					Trp81-η	2.70	2.65	11.0	2.49
**10**	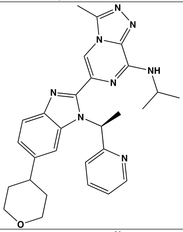	0.65	2.21<br>2.59	0.49<br>1.72	Trp81- ζ	2.57	2.44	18.3	2.60
					Trp81-η	3.02	2.78	23.0	1.45
**11**	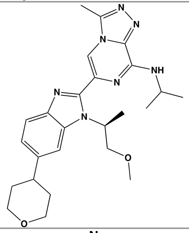	0.59	2.04	0.45	Trp81- ζ	2.52	2.45	13.5	2.97
**12**	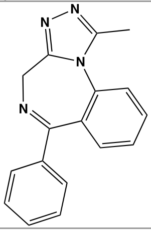	0.06	-0.05	0.23	Trp81-η	3.71	3.27	28.2	0.68
**13**	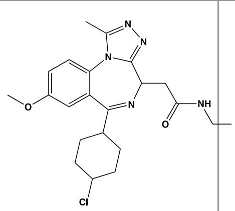	0.09	0.02	0.26	Trp81-η	3.90	2.72	45.8	0.20

Table 1: Structures, experimental ^{1}H-CSP values and geometric parameters extracted from X-Ray crystallography data listing the proton to ring-center distance (H--X), proton to aromatic plane distance (H--Y), angle (θ) between the donor proton and the ring normal through the aromatic center and Δσ the calculated change in the isotropic nuclear shielding constant for ligands 2 to 13. Ligand 1 does not form a CH-π interaction with the ligand due to a conformational rearrangement of the interacting Trp81 residue upon binding. For NMR experiments, protein concentration was 200 µM and ligand concentration 1 mM. Two sets of geometric parameters were extracted for ligand 5 corresponding to two different ligand conformations observed in the X-Ray structures. Two sets of peaks were observed for ligand 9 due to the presence of diastereoisomers. The large difference in η-CSP values for ligand 10 is likely due to a ring-flip of the pyridine engaging the η-CH group.

Experimental Considerations

We believe the major strength of this method is to provide quick and information-rich data for early screening campaigns up to later lead optimization programs. Depending on the target, fragment screens produce very few to hundreds of hits and especially in the latter case additional selection criteria regarding binding site and interaction characteristics are of great value. Also, since NMR is a solution method that reports on conformational averages, the obtained results are often a better representation of the “real” state. While NMR data is often benchmarked using X-ray structures, crystal contacts and freezing of conformers may not adequately represent the solution state. This in turn can lead to erroneous estimates of CH-π contributions to affinity. Furthermore, advanced compounds that have higher affinity may not always deliver a co-crystal structure and not every X-ray system is easily soakable, which makes NMR competitive in terms of throughput and resources. However, as with any other protein NMR system, protein size, construct selection and careful choice of experimental conditions remain critical factors.

Non-amide protein proton shifts are mostly influenced directly by through space effects and here most notably by the ring current [1]. Typical aromatic tryptophan proton shifts are in the range of 6.5 to 7.5 ppm. As exemplified in this paper, specific Trp protons experience extensive shielding upon interaction with the aromatic π-system of a ligand when involved in a favorable CH-π contact resulting in upfield shifts of up to 2.5 ppm, placing the NMR signal close to, or directly underneath the water signal at 4.7 ppm. Therefore, we conducted all experiments under complete D_2O substituted buffer conditions.

Another point to consider is the source of the CSPs. Differentiation whether the aromatic system effecting the donor-proton originates directly from the added ligand or indirectly from other nearby aromatic protein side chain that experience a change in conformation upon ligand addition is not possible from this single ^{13}C HSQC experiment. Therefore, one should include all information available to put the observed proton chemical shift into context of the system under study. This includes amino acid composition of the binding site, X-ray/modeling data, and ^{15}N HSQC data in order to detect/anticipate large conformational rearrangements in the protein under study.

The degree of ligand saturation is a crucial factor. The protein should be fully saturated with ligand in order to minimize exchange contributions from unbound protein species. Hence, high ligand solubility especially for weaker ligands found in fragment-based approaches is a prerequisite to ensure sufficiently high protein bound fraction. For example, 90% saturation of the binding site, with a 1 mM fragment would require a concentration of 10 mM. For weak ligands in fast exchange with the target, full saturation can be extrapolated by determining the CSPs as a function of ligand concentration. In cases where the requirement for protein saturation is met, the persistence of exchange phenomena can reveal important information about the bound state.

Depending on the time-scale of the process, NMR signals originating from the same nucleus can appear broadened or be split into two separate signals [2]. These phenomena can arise from bound state dynamics and local conformational heterogeneity [3], especially for less decorated ligands in earlier stages of a drug discovery process. Such information is sometimes not extractable from X-Ray structures and important for medicinal chemists when it comes to fragment growing.

General Methods

Sample Preparation

Human Brd4-BD1 (44-168) containing a TEV-cleavable His6-tag was expressed in *E. coli* BL21(DE3). 4L of bacterial cells were grown in LB to an optical density of 0.7, pelleted at 20 °C and resuspended in 1 L of M9 media supplemented with ^{15}N-labelled ammonium chloride. Expression was induced with a final concentration of 0.4mM IPTG and allowed to continue overnight at 18 °C. Cells were harvested around 18 hours after induction and subsequently resuspended in 40 mL Phosphate Buffer (20 mM sodium phosphate, 500 mM NaCl, 20 mM imidazole, pH 7.5) After sonication, the supernatant containing soluble Brd4-BD1 was loaded onto a Ni^{2+} affinity column. After binding, the protein was eluted with a step gradient of 500mM imidazole, buffer-exchanged to low imidazole Phosphate Buffer and subjected to TEV-protease cleavage overnight at 4°C. After cleaving, the protein was passed over a Ni^{2+} affinity column a second time to bind cleaved HIS6, HIS6-TEV and unselectively bound impurities. The flow-through containing human Brd4-BD1 was concentrated using a centrifugal filter device with a 3 kDa cutoff to concentrations of around 500 μM and stored at -20 °C in Phosphate Buffer containing 10 mM sodium phosphate, 100 mM NaCl (pH 7.5) 1mM DTT. For experiments under deuterated buffer conditions, Brd4-BD1 was buffer-exchanged into D_2O Phosphate Buffer containing 10 mM sodium phosphate, 100 mM NaCl (pH 7.5), 1mM DTT. To fully get rid of residual Hydrogens present in crystalline buffer components the protein was lyophilized after buffer exchange and resuspended with pure D_2O to the initial volume. The extent of residual Protons after the exchange procedure was checked with conventional 1D-^1H NMR.

Isotope Labeling

Selectively $^{13}C^2D$-tryptophan labeled Brd4-BD1 was prepared following the standard protocol additionally supplementing the media with 10 mg 3,5-dideuterio[4,6-$^{13}C_2$]anthranilic acid for the labeling of tryptophan η- and ε-carbons, or 10 mg 4,6-dideuterio[5-^{13}C]anthranilic acid for ζ-carbon labeling respectively.[4]

Protein NMR spectroscopy

All protein NMR experiments were conducted at 298 K on a Bruker Avance 600 MHz spectrometer equipped with a TCI cryoprobe with Brd4-BD1 sample concentrations of 200 μM. Ligand concentrations were 1mM for all data discussed in this work. 2D ^{1}H-^{13}C HSQC spectra were acquired using the pulse sequence 'hsqcetgpsi' of the Bruker library.[5] J_{CH} was optimized to 155 Hz. Since all experiments were conducted under fully D_2O substituted buffer conditions, the proton-channel trim-pulse was set to 0 μs to avoid unwanted excitations of the Trp resonances close to the water signal. Spectra were recorded using 64 (*t1*) x 1024 (*t2*) complex points with acquisition times of 10.6 ms (*t2*) and 65.5 ms (*t2*). A total of 32 scans were recorded per t1 increment with a recycle delay of 1.5 sec.

For Brd4-BD1, the assignment of the individual signals was carried out as follows. Trp81 signals experience CSPs upon ligand addition. Signals Trp75 and Trp119 can be distinguished given their distinct relaxation properties. Trp75 is embedded in the protein core whereas Trp119 is solvent exposed resulting in clearly different line-shapes. For systems with more Trp residues, assignment of the individual η-, ε-, and ζ-signals can be achieved using point mutations in combination with the two individual precursors: (one labeled at ζ and the other one at positions η & ε). The assignment of the ζ signals using the ζ-precursor is unambiguous, the η and ε signals labeled with the second precursor can be distinguished given their distinct difference in ^{13}C chemical shift.

NMR of small molecules

NMR experiments were recorded on Bruker Avance HD 400 or 500 MHz spectrometers equipped with Prodigy or TCI cryoprobes at 298 K, respectively. Samples were dissolved in 600 μL DMSO-d6 with TMS added as an internal standard. 1D ^{1}H spectra were acquired with 30° excitation pulses and an interpulse delay of 4.2 sec with 64k data points and 20 ppm sweep width. 1D ^{13}C spectra were acquired with the 'jmod' pulse sequence and the J_{CH} coupling constant set to 145 Hz, with broadband composite pulse decoupling (WALTZ16) and an interpulse delay of 3.3 sec with 64 k data points and a sweep width of 240 ppm. Processing and analysis of 1D spectra was performed with Bruker Topspin 3.2 software. No zero filling was performed and spectra were manually integrated after automatic baseline correction. Chemical shifts are reported in ppm on the δ scale. HSQC spectra were recorded on all samples using the pulse sequence 'hsqcetgpsi' to aid the interpretation of the data and to identify signals hidden underneath solvent peaks. Spectra were acquired with sweep widths obtained by automatic sweep width detection from 1D reference spectra in the direct dimension with 1k datapoints and with 210 ppm and 256 datapoints in the indirect dimension.

Crystallization, Processing, and Refinement of Brd4-BD1 Ligand Structures

For crystallographic studies Brd4-BD1 was concentrated to a final concentration of 11 mg/mL in a buffer containing 10 mM HEPES and 100 mM sodium chloride (pH 7.5). Brd4-BD1 was incubated with 2 mM ligand, and cocrystals in complex with ligand 1-4 were grown by mixing 1 μL of protein solution with 1 μL of reservoir solution (29 % PEG 3350 w/v, 200 mM disodium malonate, and HEPES, pH 7.2) using the hanging drop vapor diffusion method at 293 K. Crystals appeared after 4 days. Before flash-freezing in liquid nitrogen, the crystals were cryoprotected by the addition of ethylene glycol in the crystallization drop to a final concentration of 25 % (v/v). Images were processed with autoPROC [6]. The resolution limits were set using default autoPROC and STARANISO [7] settings. The structures were solved by molecular replacement using the Brd4-BD1 structure 2OSS as a search model. Subsequent model building and refinement were done using standard protocols using CCP4 [8], COOT [9] and autoBUSTER [10]. Crystallographic data collection statistics and refinement statistics can be found in supplementary tables 2-5. Crystal structures and structure factors were deposited at the PDB with the accession codes 6XVC (ligand 1), 6XV7 (ligand 2), 6XV3 (ligand 3) and 6XUZ (ligand4).

Crystallographic data collection statistics and refinement statistics

Table 2: Ligand 1-Data collection and refinement statistics

	Ligand 1
**Data collection***	
Space group	P212121
Cell dimensions	
a, *b*, *c* (Å)	39.20, 50.67, 57.54
a, b, g (°)	90.0, 90.0, 90.0
Resolution (Å)	1.10 (1.16-1.10)
R_{merge}	6.1 (124.8)
CC (1/2)	0.999 (0.629)
I / s*I*	13.4 (1.4)
Completeness (spherical, %)	84.3 (28.1)
Completeness (ellipsoidal, %)	90.9 (46.1)
Redundancy	6.4 (6.4)
**Refinement****	
Resolution (Å)	1.10
No. reflections	39689
R_{work} / R_{free}	17.2/19.8
No. atoms	
Protein	1043
Ligand/ion	37
Water	115
B-factors	
Protein	14.37
Ligand/ion	20.85
Water	26.99
R.m.s. deviations	
Bond lengths (Å)	0.010
Bond angles (°)	0.92

*Values in parentheses are for highest-resolution shell, values as output by STARANISO
**Values as output by autoBUSTER

Table 3: Ligand 2-Data collection and refinement statistics

	Ligand 2
**Data collection***	
Space group	P212121
Cell dimensions	
a, *b*, *c* (Å)	32.02, 47.54, 78.93
a, b, g (°)	90.0, 90.0, 90.0
Resolution (Å)	40.73-1.67 (1.75-1.67)
R_{merge}	8.0 (40.4)
CC (1/2)	0.998 (0.806)
I / s*I*	14.9 (1.8)
Completeness (spherical, %)	88.5 (36.6)
Completeness (ellipsoidal, %)	90.1 (40.4)
Redundancy	5.6 (1.6)
**Refinement****	
Resolution (Å)	1.67
No. reflections	12909
R_{work} / R_{free}	17.2/20.3
No. atoms	
Protein	1053
Ligand/ion	33
Water	125
B-factors	
Protein	11.64
Ligand/ion	17.19
Water	28.93
R.m.s. deviations	
Bond lengths (Å)	0.008
Bond angles (°)	0.84

*Values in parentheses are for highest-resolution shell using STARANISO
**Values as output by autoBUSTER

[AU: Equations defining various *R*-values are standard and hence are no longer defined in the footnotes.]
[AU: Ramachandran statistics should be in Methods section at the end of Refinement subsection.]
[AU: Wavelength of data collection, temperature and beamline should all be in Methods section.]

Table 4: Ligand 3-Data collection and refinement statistics

	Ligand 3
**Data collection***	
Space group	P1
Cell dimensions	
a, *b*, *c* (Å)	42.18, 58.96, 59.64
a, b, g (°)	105.25, 90.70, 97.94
Resolution (Å)	1.47 (1.59-1.47)
R_{merge}	5.2 (51.8)
CC (1/2)	0.998 (0.596)
I / s*I*	9.3 (1.5)
Completeness (spherical, %)	62.0 (14.5)
Completeness (ellipsoidal, %)	82.1 (62.9)
Redundancy	1.8 (1.9)
**Refinement****	
Resolution (Å)	1.47
No. reflections	57389
R_{work} / R_{free}	25.9/29.0
No. atoms	
Protein	4236
Ligand/ion	152
Water	668
B-factors	
Protein	18.54
Ligand/ion	14.58
Water	27.95
R.m.s. deviations	
Bond lengths (Å)	0.008
Bond angles (°)	0.87

*Values in parentheses are for highest-resolution shell, values as output by STARANISO
**Values as output by autoBUSTER

Table 5: Ligand 4-Data collection and refinement statistics

	Ligand 4
**Data collection***	
Space group	P212121
Cell dimensions	
a, *b*, *c* (Å)	41.86, 48.29, 57.86
a, b, g (°)	90.0, 90.0, 90.0
Resolution (Å)	37.08-1.07 (1.14-1.07)
R_{merge}	11.3 (144.9)
CC (1/2)	0.999 (0.664)
I / s*I*	10.7 (1.7)
Completeness (spherical, %)	76.4 (21.4)
Completeness (ellipsoidal, %)	91.3 (71.3
Redundancy	8.4 (9.9)
**Refinement****	
Resolution (Å)	1.07
No. reflections	4107
R_{work} / R_{free}	17.7/20.1
No. atoms	
Protein	1062
Ligand/ion	35
Water	220
B-factors	
Protein	13.30
Ligand/ion	11.98
Water	24.33
R.m.s. deviations	
Bond lengths (Å)	0.010
Bond angles (°)	0.95

*Values in parentheses are for highest-resolution shell, values as output by STARANISO
**Values as output by autoBUSTER

DFT calculations

We employed dispersion corrected density functional theory at wB97XD/cc-pVTZ level of theory [11] to estimate CH-π interactions between two orthogonal benzene molecules using Gaussian09 revision D.01 [12]. This calculation level has been validated earlier to reproduce energies from post-Hartree-Fock calculations at significantly lower computational cost [13]. Therefore, we individually optimized the benzene monomers in vacuum. Subsequently, we identified an optimal height of 2.5 Å for the interacting hydrogen of the second benzene over the ring centroid via a distance scan along the orthogonal of the benzene plain in 0.1 Å steps. This height was held constant for subsequent scans of benzene-benzene interactions in 0.2 Å steps

either along the CH bond vectors (x-axis) or between those (y-axis). The Counterpoise method was employed to correct for basis set superposition error.

ITC

Calorimetric titrations were measured at 25 °C on a MicroCal PEAQ-ITC (Malvern) instrument. The sample cell contained a solution of 18 μM Brd4-BD1 in a buffer consisting of 10 mM sodium phosphate, 100 mM NaCl (pH 7.5), 1mM DTT. Brd4-BD1 ligands were dissolved in the same buffer at a 150 -250 μM concentration and loaded into the injection syringe. Titration curves were generated by 18 successive injections of 2 μL Brd4-BD1 ligands spaced at 150 s intervals. ITC experiments were performed in duplicates and the data was analyzed using the MicroCal PEAQ-ITC Analysis Software (Malvern). The dissociation constant (K_D) and the stoichiometry (N) were calculated by fitting the thermograms to one binding site.

RP-HPLC

Preparative RP-HPLC purification was achieved on Agilent or Gilson systems using columns from Waters (Sunfire C18 OBD, 5 or 10 μm, 20 x 50 mm, 30 x50 mm or 50 x 150 mm; X-Bridge C18 OBD, 5 or 10 μm, 20 x 50, 30 x 50, or 50 x 150 mm) or YMC (Triart C18, 5 or 10 μm, 20 x 50 mm, or 30 x 50 mm). Compounds were eluted with MeCN/water gradients using either acidic (0.2 % HCOOH or TFA) or basic water (5 mL 2 M NH_4HCO_3 + 2 mL NH_3 (32 %) made up to 1 L with water).

HRMS

HRMS data was recorded using a LTQ Orbitrap XL (Thermo Scientific) coupled with a Triversa Nanomate Nanospray ion source (ADVION Bioscience Inc.) The mass calibration was performed using the Pierce LTQ Velos ESI positive ion calibration solution from Thermo Scientific (Product Nr. 88323).

MS parameters:

The scan window was set to 50–400 amu with a maximum injection time of 500 ms and 1 microscan. Resolution of the Orbitrap was 60000 with a mass accuracy ≤ 5 ppm. The ion mode set to positive with a capillary temperature 200 °C and voltage of 60 eV. The tube lens potential was set to 110 eV.

NanoESI voltage was 1.45 kV and the N_2-gas pressure set to 0.45 psi. Total sample volume was 5 µl and the acquisition time was 0.4 sec, with 10 scans of averaging per spectrum Sample dilution: 10 mM DMSO stock solution was diluted 1:200 in 50 % MeOH +0.01 % formic acid.

Synthetic Protocols

Synthesis of ligand 1

(*S*)-3-((*R*)-1-((7-(3-methyl-[1,2,4]triazolo[4,3-*a*]pyridin-6-yl)quinolin-5-yl)oxy)ethyl)pyrrolidin-2-one

The synthesis of compound **(1)** is covered in patent WO 2013014060. 3-methyl-6-(4,4,5,5-tetramethyl-1,3,2-dioxaborolan-2-yl)-[1,2,4]triazolo[4,3-*a*]pyridine **(2)** was purchased from commercial sources. **(1)** (40 mg, 107 µmol), **(2)** (34 mg, 129 µmol), sodium carbonate (23 mg, 215 µmol)and Pd tetrakis (13 mg, 11 µmol) were dissolved in 100 µl water and 500 µl dioxane and stirred at 140 °C for 15 min in the microwave resulting in a complete conversion of the educts according to HPLC/MS. The reaction mixture was purified via semi-preparative HPLC (SunFire, acidic, MeOH, 60 °C). Fractions containing **ligand 1** were pooled and freeze-dried. (yield: 15 mg; 36 %)

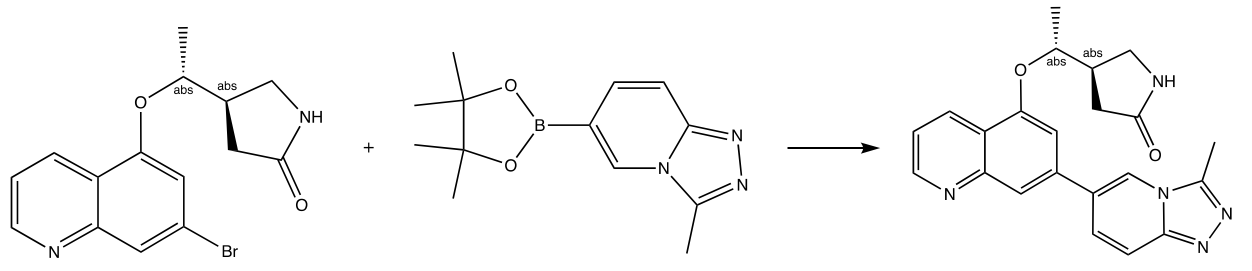

(1) (2) Ligand 1

^{1}H NMR (500 MHz, DMSO-d$_6$) δ 8.88 (dd, *J*=1.60, 4.10 Hz, 1H), 8.71 (s, 1H), 8.46 (dd, *J*=1.00, 8.50 Hz, 1H), 7.95 (s, 1H), 7.83 (dd, *J*=1.60, 9.60 Hz, 1H), 7.78 (dd, *J*=0.90, 9.60 Hz, 1H), 7.56 (s, 1H), 7.47 (dd, *J*=4.10, 8.50 Hz, 1H), 7.40 (br s, 1H), 4.93 (quin, *J*=5.91 Hz, 1H), 3.38 (t, *J*=9.14 Hz, 1H), 3.11 (dd, *J*=6.31, 9.77 Hz, 1H), 2.76-2.85 (m, 1H), 2.74 (s, 3H), 2.30-2.37 (m, 1H), 2.20-2.27 (m, 1H), 1.30 (d, *J*=5.99 Hz, 3H)

^{13}C NMR (126 MHz, DMSO-d$_6$) δ 176.6, 153.9, 152.0, 149.3, 148.9, 144.8, 137.9, 130.8, 128.3, 126.2, 122.3, 121.5, 120.9, 119.5, 115.6, 106.6, 76.3, 44.1, 40.6, 32.9, 17.3, 10.5

HPLC t_R =1.19; HPLC purity: >95 %

HRMS (*m/z*): $[M+H]^+$ calcd. for $C_{22}H_{21}N_5O_2$, 388.17678; found, 388.17563

Synthesis of ligand 2

***N*-(3,4-difluorobenzyl)-*N*,3-dimethyl-[1,2,4]triazolo[4,3-*b*]pyridazin-6-amine**

6-chloro-3-methyl-[1,2,4]triazolo[4,3-*b*]pyridazine **(3)** and 1-(3,4-difluorophenyl)-*N*-methylmethanamine **(4)** were purchased from commercial sources. To **(3)** (60 mg; 0,356 mmol) was added NMP (600 µl), **(4)** (0,074 ml; 0,534 mmol) and DIPEA (0,075 ml; 0,464 mmol) and stirred at 120 °C o/n. Purification was achieved on a Gilson system using a column of Waters (XBridge Prep C18 OBD; 10 µm; 30*100 mm). Compounds were eluted with a MeCN/water gradient using basic water (10 mM NH_4HCO_3, 38 mM NH_3. Product fractions containing **ligand 2** were pooled and freeze-dried. (yield: 31 mg; 30,1 %)

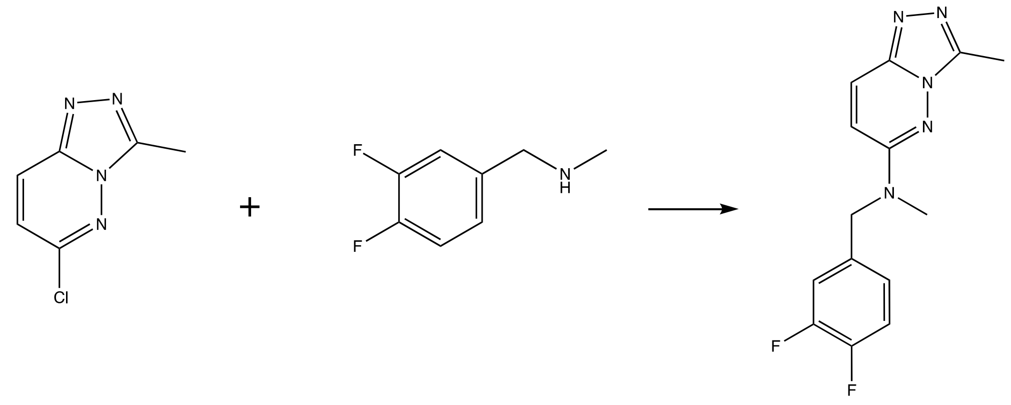

(3) (4) Ligand 2

^{1}H NMR (500 MHz, DMSO-d_6) δ 8.01 (d, *J*=10.09 Hz, 1H), 7.36-7.42 (m, 2H), 7.22 (d, *J*=10.09 Hz, 1H), 7.14-7.18 (m, 1H), 4.78 (s, 2H), 3.16 (s, 3H), 2.54 (s, 3H)

^{13}C NMR (125 MHz, DMSO-d_6) δ 154.7, 149.9, 149.0, 145.6, 142.3, 136.1, 124.7, 124.5, 118.0, 116.9, 113.6, 52.8, 37.3, 9.7

HPLC t_R =1.02; HPLC purity: >95 %

HRMS (*m/z*): $[M+H]^+$ calcd. for $C_{14}H_{13}F_2N_5$, 290.12118 ; found, 290.12187

Synthesis of ligand 3

***N*-isopropyl-3-methyl-6-(6-((*S*)-3-methylmorpholino)-1-((*S*)-1-phenylethyl)-3a,7a-dihydro-1*H*-imidazo[4,5-*c*]pyridin-2-yl)-[1,2,4]triazolo[4,3-*a*]pyrazin-8-amine**

Synthesis of the staring material **(5)** and **(6)** is described in WO2014076237. 8-Isopropylamino-3-methyl-[1,2,4]triazolo[4,3-a]pyrazine-6-carboxylic acid (50 mg; 0.213 mmol) is solved in 3 ml Dichloromethane and treated with HATU (122 mg; 0.319 mmol). Starting material **(6)** (66 mg; 0.213 mmol) is added. The reaction mixture is stirred for 1 h at room temperature, then diluted with DCM and extracted with half saturated $NaHCO_3$ solution. The organic layer is dried over $MgSO_4$ and evaporated to dryness. This intermediate is dissolved in 2.5 ml acetic acid and heated in a microwave reactor to 180 °C for 2 h. Afterwards the solvents are evaporated and the crude product purified on a Gilson system using a column of Waters (XBridge Prep C18 OBD; 10 µm; 30*100 mm). Compounds were eluted with a MeCN/water gradient using basic water (10 mM NH_4HCO_3, 38 mM NH_3 yielding 50 mg of the title compound (45 %; 0.098 mmol).

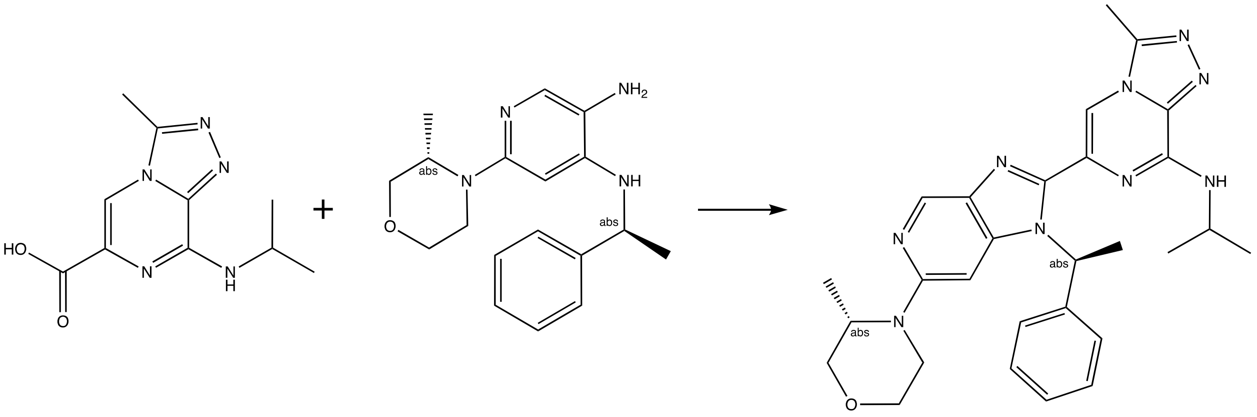

(5) (6) Ligand 3

^{1}H NMR (400 MHz, DMSO-d$_6$) δ 8.59 (s, 1H), 8.30 (s, 1H), 8.20 (d, *J*=8.11 Hz, 1H), 7.38-7.42 (m, 2H), 7.33-7.38 (m, 2H), 7.27-7.32 (m, 1H), 6.91 (q, *J*=7.10 Hz, 1H), 6.12 (s, 1H), 4.09-4.21 (m, 2H), 3.89 (br dd, *J*=3.04, 10.90 Hz, 1H), 3.64-3.70 (m, 1H), 3.53-3.62 (m, 2H), 3.46 (dt, *J*=3.70, 12.30 Hz, 1H), 2.80 (dt, *J*=3.68, 12.23 Hz, 1H), 2.73 (s, 3H), 1.95 (d, *J*=7.35 Hz, 3H), 1.14 (t, *J*=6.21 Hz, 6H), 0.98 (d, *J*=6.59 Hz, 3H)

^{13}C NMR (100 MHz, DMSO-d$_6$) δ 154.5, 150.3, 147.2, 146.1, 142.2, 140.1, 140.0, 138.7, 134.7, 131.7, 128.9, 127.9, 127.1, 108.0, 88.7, 71.1, 66.6, 54.1, 47.9, 42.4, 40.6, 22.2, 17.8, 12.0, 10.2

HPLC t_R =1.31; HPLC purity: >95 %

HRMS (*m/z*): $[M+H]^+$ calcd. for $C_{28}H_{33}N_9O$, 512.28808; found, 512.28781

Synthesis of ligand 4

***N*-isopropyl-6-(1-((*S*)-1-methoxypropan-2-yl)-6-((*S*)-3-methylmorpholino)-3a,7a-dihydro-1*H*-imidazo[4,5-*c*]pyridin-2-yl)-3-methyl-[1,2,4]triazolo[4,3-*a*]pyrazin-8-amine**

Synthesis of the staring material **(5)** and **(6)** is described in WO2014076237 and WO2015067770. 8-Isopropylamino-3-methyl-[1,2,4]triazolo[4,3-a]pyrazine-6-carboxylic acid (80 mg; 0.340 mmol) is solved in 5 ml Dichloromethane and treated with HATU (190 mg; 0.500 mmol). Starting material **(6)** (95 mg; 0.340 mmol) is added. It is stirred for 1 h at room temperature. The reaction mixture is diluted with DCM and extracted with half saturated NaHCO3 solution. The organic layer is dried over MgSO4 and evaporated to dryness. This intermediate is dissolved in 2.5 ml acetic acid and heated in a microwave reactor to 180 °C for 2 h. Afterwards the solvents are evaporated and the crude product purified on a Gilson system using a column of Waters (XBridge Prep C18 OBD; 10 µm; 30*100 mm). Compounds were eluted with a MeCN/water gradient using basic water (10 mM NH_4HCO_3, 38 mM NH_3 yielding128 mg of the title compound (79 %; 0.268 mmol).

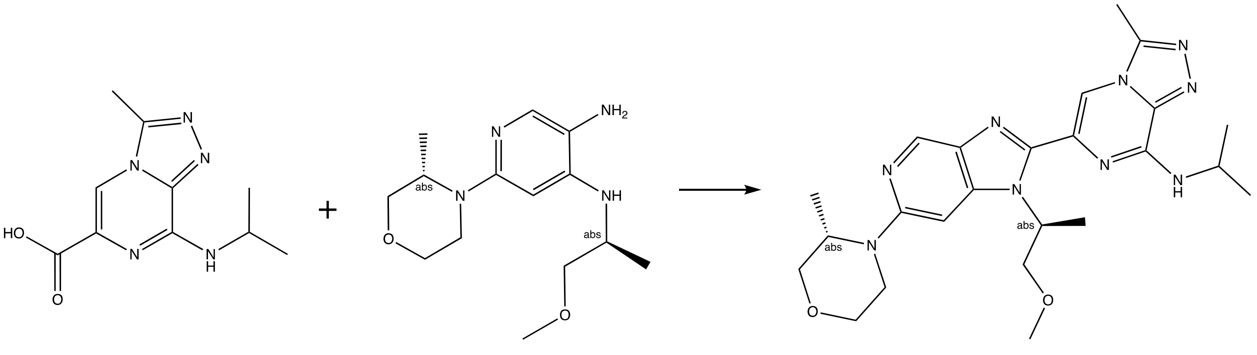

(5) (7) Ligand 4

^{1}H NMR (500 MHz, DMSO-d_6) δ 8.59 (s, 1H), 8.21 (s, 1H), 8.23 (d, *J*=8.83 Hz, 1H), 6.85 (s, 1H), 5.68 (sxt, *J*=7.00 Hz, 1H), 4.33-4.52 (m, 2H), 3.92-4.05 (m, 2H), 3.82 (br d, *J*=12.61 Hz, 1H), 3.67-3.78 (m, 3H), 3.55 (dt, *J*=2.99, 11.59 Hz, 1H), 3.14 (s, 3H), 3.08 (dt, *J*=3.63, 12.37 Hz, 1H), 2.71 (s, 3H), 1.61 (d, *J*=6.94 Hz, 3H), 1.30 (t, *J*=5.99 Hz, 6H), 1.10 (d, *J*=6.62 Hz, 3H)

^{13}C NMR (125 MHz, DMSO-d_6) δ 154.9, 150.6, 147.1, 146.0, 142.4, 139.9, 138.6, 134.6, 131.9, 107.9, 88.9, 73.1, 71.3, 66.8, 58.6, 52.5, 47.9, 42.5, 40.9, 22.4, 22.3, 16.0, 12.0, 10.2

HPLC t_R =1.16; HPLC purity: >95 %

HRMS (*m/z*): $[M+H]^+$ calcd. for $C_{24}H_{33}N_9O_2$, 480.28299; found, 480.28320

References:

[1] M. P. Williamson, *Prog Nucl Magn Reson Spectrosc* **2013**, *73*, 1-16.

[2] A. G. Palmer, *Chemical Reviews* **2004**, *104*, 3623-3640.

[3] a) D. L. Mobley, K. A. Dill, *Structure* **2009**, *17*, 489-498; b) C. E. Chang, W. Chen, M. K. Gilson, *Proc Natl Acad Sci U S A* **2007**, *104*, 1534-1539; c) A. B. Sahakyan, M. Vendruscolo, *J Phys Chem B* **2013**, *117*, 1989-1998; d) G. C. P. van Zundert, B. M. Hudson, S. H. P. de Oliveira, D. A. Keedy, R. Fonseca, A. Heliou, P. Suresh, K. Borrelli, T. Day, J. S. Fraser, H. van den Bedem, *Journal of Medicinal Chemistry* **2018**, *61*, 11183-11198.

[4] J. Schörghuber, L. Geist, M. Bisaccia, F. Weber, R. Konrat, R. J. Lichtenecker, *Journal of Biomolecular NMR* **2017**, *69*, 13-22.

[5] a) A. G. Palmer, J. Cavanagh, P. E. Wright, M. Rance, *Journal of Magnetic Resonance (1969)* **1991**, *93*, 151-170; b) L. Kay, P. Keifer, T. Saarinen, *Journal of the American Chemical Society* **1992**, *114*, 10663-10665; c) J. Schleucher, M. Schwendinger, M. Sattler, P. Schmidt, O. Schedletzky, S. J. Glaser, O. W. Sorensen, C. Griesinger, *J Biomol NMR* **1994**, *4*, 301-306.

[6] C. Vonrhein, C. Flensburg, P. Keller, A. Sharff, O. Smart, W. Paciorek, T. Womack, G. Bricogne, *Acta Crystallographica Section D* **2011**, *67*, 293-302.

[7] I. J. Tickle, Flensburg, C., Keller, P., Paciorek, W., Sharff, A., Vonrhein, C., Bricogne, G., *Cambridge, United Kingdom: Global Phasing Ltd.* **2018**.

[8] M. D. Winn, C. C. Ballard, K. D. Cowtan, E. J. Dodson, P. Emsley, P. R. Evans, R. M. Keegan, E. B. Krissinel, A. G. Leslie, A. McCoy, S. J. McNicholas, G. N. Murshudov, N. S. Pannu, E. A. Potterton, H. R. Powell, R. J. Read, A. Vagin, K. S. Wilson, *Acta Crystallogr D Biol Crystallogr* **2011**, *67*, 235-242.

[9] P. Emsley, B. Lohkamp, W. G. Scott, K. Cowtan, *Acta Crystallogr D Biol Crystallogr* **2010**, *66*, 486-501.

[10] B. E. Bricogne G., Brandl M., Flensburg C., Keller P., Paciorek W.,, S. A. Roversi P, Smart O.S., Vonrhein C., Womack T.O., *Cambridge, United Kingdom: Global Phasing Ltd.* **2017**.

[11] J.-D. Chai, M. Head-Gordon, *Physical Chemistry Chemical Physics* **2008**, *10*, 6615-6620.

[12] G. W. T. M. J. Frisch, H. B. Schlegel, G. E. Scuseria, M. A. Robb, J. R. Cheeseman, G. Scalmani, V. Barone, G. A. Petersson, H. Nakatsuji, X. Li, M. Caricato, A. Marenich, J. Bloino, B. G. Janesko, R. Gomperts, B. Mennucci, H. P. Hratchian, J. V. Ortiz, A. F. Izmaylov, J. L. Sonnenberg, D. Williams-Young, F. Ding, F. Lipparini, F. Egidi, J. Goings, B. Peng, A. Petrone, T. Henderson, D. Ranasinghe, V. G. Zakrzewski, J. Gao, N. Rega, G. Zheng, W. Liang, M. Hada, M. Ehara, K. Toyota, R. Fukuda, J. Hasegawa, M. Ishida, T. Nakajima, Y. Honda, O. Kitao, H. Nakai, T. Vreven, K. Throssell, J. A. Montgomery, Jr., J. E. Peralta, F. Ogliaro, M. Bearpark, J. J. Heyd, E. Brothers, K. N. Kudin, V. N. Staroverov, T. Keith, R. Kobayashi, J. Normand, K. Raghavachari, A. Rendell, J. C. Burant, S. S. Iyengar, J. Tomasi, M. Cossi, J. M. Millam, M. Klene, C. Adamo, R. Cammi, J. W. Ochterski, R. L. Martin, K. Morokuma, O. Farkas, J. B. Foresman, and D. J. Fox, *Gaussian, Inc., Wallingford CT* **2016**.

[13] R. G. Huber, M. A. Margreiter, J. E. Fuchs, S. von Grafenstein, C. S. Tautermann, K. R. Liedl, T. Fox, *Journal of Chemical Information and Modeling* **2014**, *54*, 1371-1379.
